# The Influence of the COVID-19 Pandemic on Social Anxiety: A Systematic Review

**DOI:** 10.3390/ijerph20032362

**Published:** 2023-01-29

**Authors:** Reuben Kindred, Glen W. Bates

**Affiliations:** Department of Psychological Sciences, Swinburne University of Technology, John St, Hawthorn, VIC 3122, Australia

**Keywords:** coronavirus, COVID-19, general population, lockdown, pandemic, prevalence, anxiety, social anxiety, social phobia, systematic review

## Abstract

The COVID-19 pandemic has resulted in negative mental health outcomes throughout the world, and its impact on social interactions and relationships is likely to be evident in problematic social anxiety. This systematic review qualitatively synthesized data from studies that have reported on the effects of the pandemic on social anxiety. A systematic search of Web of Science Core Collection, Embase, PsychINFO, Scopus, EBSCOhost, Cochrane Central Register of Controlled Trials, and Proquest Central—Dissertations and Theses was conducted, with thirty-three studies meeting the inclusion criteria. The results suggest that social anxiety has been heightened in the general population due to the pandemic, with women and low-income earners being especially vulnerable. Other contributing factors include impaired coping strategies, lower socio-emotional well-being, limited support networks, and contraction of the SARS-CoV-2 virus. Individuals with a Social Anxiety Disorder diagnosis may be at risk of a deterioration of mental health in general. Limitations of the literature reviewed include the predominance of cross-sectional study designs, which limit causal inferences are limited. Additionally, associations may be inflated as many studies have not accounted for mediating variables. Taken together, the research suggests that social anxiety, either pre-pandemic or arising due to the pandemic environment, has contributed to a variety of negative mental health outcomes related to social anxiety.

## 1. Introduction

The COVID-19 pandemic has led to acute changes in daily routines and lifestyles worldwide, with the social consequences being detrimental to mental health [1]. For instance, there has been an estimated additional 76.2 million cases of anxiety disorders globally, an increase of 25.6% [2], and it is unlikely that mental health will recover to pre-pandemic levels for some time [3]. Several unique social phenomena have emerged within the pandemic environment. These include government-enforced quarantine and lockdowns, work and school closures, physical distancing, and avoidance of non-essential social interactions. As problematic social anxiety is characterised by intense anxiety in social interactions (e.g., meeting unfamiliar people) and is maintained through a reciprocal relationship between avoidance and fear [4], enforced social avoidance is likely to exacerbate social anxiety within community and clinical populations. Notably, despite finding social interactions stressful, socially anxious people are happier interacting with others than being alone [5] and experience loneliness when isolated from others [6]. Thus, enforced social avoidance both reinforces avoidance and deprives socially anxious people of a major source of well-being.

The pandemic is an ongoing global stressor, and as social norms are potential contributors to social anxiety [7], the resulting social isolation is likely to contribute to heightened social anxiety. Several negative mental health outcomes have been reported in healthy populations across the globe, for example, elevated levels of anxiety, stress, depression, somatic symptoms, and poor sleep quality [8,9,10,11,12,13]. Several risk and protective factors have been identified for general psychological health during the pandemic [14,15,16]. These include coping skills, psychological resilience, and social support. However, the influence of these factors is likely to vary depending on the specific form of psychopathology [17]. 

People with an established diagnosis of Social Anxiety Disorder (SAD) may be particularly vulnerable to negative mental health outcomes. The fifth edition of the Diagnostic and Statistical Manual of Mental Disorders- Technical Revision (DSM-5-TR) indicates that the core component of SAD is a “marked, or intense, fear or anxiety of social situations in which the individual may be scrutinized by others” [18], and can be triggered by a threat to social relationships [19,20]. The key fear is a negative evaluation by others, and social situations create intense and unrelenting anxiety that leads to avoidance [18]. The COVID-19 pandemic is likely to have exacerbated risk factors for SAD, especially the experience of low levels of social support [21,22] and avoidance of social situations [23]. SAD significantly interferes with the interpersonal relationships and social life of individuals [24] and is characterised by an impaired ability to function in daily life [25]. For example, school dropout and employment disruption are common in SAD. Data captured by the Australian Bureau of Statistics [26] during the pandemic indicates that, in Australia, SAD had the highest 12-month prevalence estimate of any disorder at 7.0%; previously, the 12-month prevalence estimate was 4.7%. Even prior to the onset of COVID-19, SAD has been increasingly recognised as a hidden epidemic [27]. This suggests that there may be a greater incidence of SAD due to the COVID-19 pandemic. Furthermore, individuals with SAD or heightened social anxiety may be an especially vulnerable population in the pandemic. Relatedly, how pre-pandemic social anxiety directly influences affective, behavioural, and cognitive responses to the COVID-19 environment has important implications for treatment.

There have been reviews that have revealed the impacts of the COVID-19 pandemic on mental health in general [28,29,30,31]. Several stress factors, such as educational institution closures, financial strain, unemployment, and a sense of unpredictability, may enhance the risk of negative mental health outcomes [1,32,33]. For instance, Kan et al. found a moderately high rate of prevalence of anxiety for both the infected and non-infected populations [34]. To date, research on social anxiety in the context of the pandemic has not been reviewed. Considering the unique social features of the COVID-19 environment, this is unexpected. To our understanding, this review is novel in focusing on the influence of the COVID-19 pandemic on social anxiety. By extension, it is important to discern how social anxiety is related to other mental health constructs as many domains have been affected (e.g., social, educational, occupational); it is likely that individuals have experienced multifaceted sequelae in the ongoing wake of the pandemic. We decided, therefore, to systematically review the existing studies in this field with a view to providing a holistic approach, allowing future research to focus on unaddressed issues. The current systematic review had four aims. First, to assess whether the incidence of social anxiety has increased within the general population because of the COVID-19 pandemic. Second, to determine how COVID-19 has affected individuals with a clinical diagnosis of Social Anxiety Disorder. Third, to ascertain which risk and protective factors may have influenced levels of social anxiety in the context of the COVID-19 pandemic. Fourth, to determine how social anxiety has influenced affective, behavioural, and cognitive responses to the COVID-19 environment.

## 2. Methods

### 2.1. Protocol and Registration

This systematic review followed the guidelines of Preferred Reporting Items for Systematic Reviews and Meta-analyses (PRISMA). The protocol was registered with PROSPERO ID: CRD42021275910. The PRISMA checklist is located in Appendix A. 

### 2.2. Search Strategy and Study Selection

A systematic search was conducted from inception to 1 September 2021 and then rerun on 1 March 2022, 1 August 2022, and 1 November 2022. The following databases were searched: Web of Science Core Collection, Embase, PsychINFO, Scopus, EBSCOhost, Cochrane Central Register of Controlled Trials, and Proquest Central—Dissertations and Theses.

The search terms were (1) “Social Anxiety” OR “Social phob*”, AND (2) “COVID*” OR “Coronavirus” OR “Pandemic” OR “Lockdown”. We did not include “SARS-CoV-2” as a search term. Not all systematic reviews have included this term, e.g., [35], and as including the search term “SARS-CoV-2” yields few extra hits, the results of the review are unlikely to be affected. No limiters were applied to the search. Details of the search strategy are supplied in Appendix B. As the current systematic review attempts to answer multiple clinical questions relating to prognosis, prevention, and etiology; as such, PICO components address each research question (Appendix C). The records were imported into Endnote X9, the duplicates were removed via EndNote’s duplicate identification strategy, and the titles and abstracts were then screened by both authors. To be included, studies needed to have (1) assessed either the prevalence or effect of social anxiety on responses to the COVID-19 pandemic; (2) included empirical data; and (3) been published in English. Studies were excluded if they were a (1) study protocol or (2) if the abstracts and/or full texts were unavailable. The full-text publications were then screened for eligibility by both authors, and disagreements were resolved though discussion. 

### 2.3. Search Results

**Figure 1:**
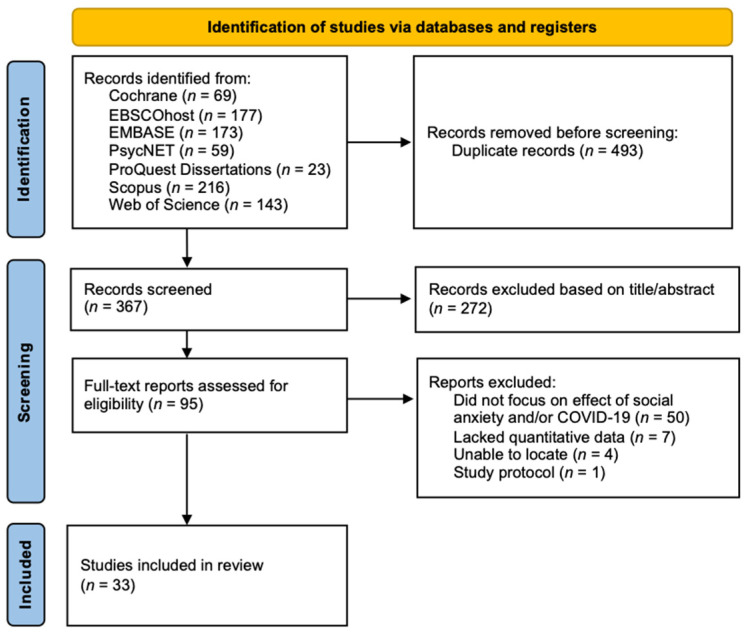
PRISMA Flow-chart for the Selection of Eligible Studies.

The title and abstracts of 860 studies found via database searches were extracted during the search. After duplicate removal, title and abstract screening, and full-text screening based on the eligibility criteria, a total of thirty-three studies were included in the final analysis. Figure 1 provides a PRISMA chart summarizing the review process. 

### 2.4. Data Extraction

The data were extracted with a data extraction template in Microsoft Office Excel. The information extracted from the studies included the following: country, sample size, gender and age characteristics of the sample, type of population, study design, date of data collection, and relevant measures implemented in the studies.

### 2.5. Study Quality Assessment

The QualSyst tool [36] was employed to determine study quality. Study quality was assessed dependently: both authors discussed studies and agreed on scores on each criterion. Regarding quantitative studies, QualSyst establishes a study quality coefficient based on fourteen criteria. The QualSyst summary score is the total score divided by the total score possible sum (i.e., inapplicable criteria do not affect the summary score), with possible scores ranging from 0.0 to 1.0. It is suggested [37] that QualSyst scores can be interpreted as follows: limited (<0.50), adequate (0.50–0.70), good (0.71–0.79), and strong (>0.80).

### 2.6. Data Synthesis

The searches identified a number of study designs and a multitude of various outcomes; therefore, a meta-analysis was not feasible for this study design. As such, the results of the studies were summarised in a narrative manner. Whilst effect sizes were not synthesized, they were calculated to allow for the streamlined interpretation of the results. To assess the incidence of social anxiety within the general population, a Standardised Mean Difference (SMD) was calculated for appropriate statistics if a study had not already been conducted. If a study did not report means and standard deviations, alternative statistics (e.g., *t*-value, *p*-value) were used to calculate Cohen’s d. Calculations of weighted mean effect sizes were conducted with the *metafor* package version 3.0-2 [38] in version 4.0.3 of R [39]. As Cohen’s *d* belongs to the SMD effect size family, it is commonly interpreted as small (*d* = 0.2), medium (*d* = 0.5), and large (*d* = 0.8) based on benchmarks suggested by Cohen [40]. However, these interpretations should be considered arbitrary [41].

## 3. Results and Discussion

### 3.1. Summary Descriptions

The studies originated from Australia, the USA, Germany, Turkey, Lebanon, China, Poland, Malaysia, Spain, India, and Israel and were published between 2020 and 2022. The total sample sizes of the publications ranged from 32 to 3137. The mean ages of participants in trials ranged from 10.22 to 42.72 years. Data from 16,013 relevant participants were synthesized across the included studies. All studies were observational; twenty studies were cross-sectional, two were repeated cross-sectional—in which the same information is asked to an independent sample at each wave—and eleven were longitudinal. One study was a natural experiment. The authors were contacted if their studies contained data that may have been relevant to the review but not otherwise included (*k* = 2, response rate = 100%). As such, unpublished data from two studies [42,43] were included. Study characteristics and the QualSyst summary scores are supplied in Table 1.

### 3.2. Methodological Quality

The QualSyst establishes a study quality coefficient based on fourteen criteria; however, as no studies were Randomised Controlled Trials, the criteria relating to random allocation, blinding of investigators, and blinding of subjects were not applicable. The thirty-three studies obtained quality summary scores ranging from 0.41 to 0.95, out of a maximum of 1.0, and the average summary score was 0.84 (*SD* = 0.13). 

### 3.3. Social Anxiety in the General Population Due to the COVID-19 Pandemic

A small increase in social anxiety has been observed across a range of adult populations from pre-pandemic to post-pandemic, with all Cohen’s d results being positively valued (Range: 0.11 to 1.20). However, there is no evidence that lockdowns have unduly affected social anxiety levels.

Seven studies have attempted to assess changes in social anxiety due to the pandemic in adults. In Thompson et al. [42], using a retrospective measure of social anxiety in adults, social anxiety symptoms increased after the onset of the COVID-19 pandemic (*d* = 0.30, *p* = 0.001). In Liang et al. [63], a cross-sectional study, Chinese students were well above the national norm on the SAD scale (*d* = 1.20), with the difference being statistically significant (*p* < 0.001). In Langhammer et al. [61], clinical outpatients were asked about perceived changes in social anxiety due to the pandemic, but the increase was not significant (at *p* > 0.05), which is likely due to the small sample size. These findings are supported by longitudinal studies. Hawes et al. [54] reported an increase in social anxiety (*d* = 0.16), and social anxiety scores increased from pre-pandemic to post-pandemic in a study by Juvonen et al. (*d* = 0.11) [59]. There is also evidence that social anxiety has varied throughout the pandemic itself. For instance, Lim et al. [64] found that social anxiety increased over the first six months of the pandemic (*β* = 0.65, *p* < 0.001). Regarding social anxiety levels during lockdowns, a longitudinal study by Buckner et al. [47] found no statistically significant change in social anxiety for young adults before and during a lockdown (*d* = −0.07).

The impact of the pandemic on social anxiety in children and adolescent populations was assessed in four studies, with all Cohen’s *d* results indicating an increase in social anxiety (Range: 0.06 to 0.26). One cross-sectional study [72] found that scores in a parent-reported social anxiety measure were greater for the pandemic period than the retrospectively assessed pre-pandemic period (*d* = 0.25), reaching statistical significance (*p* < 0.001). In a longitudinal study by Charmaraman et al. [49], there was a statistically significant (*p* < 0.001) increase in social anxiety from pre-pandemic to post-pandemic (*d* = 0.26). Two other longitudinal studies reported statistically non-significant increases in social anxiety. In Yurteri & Sarigedik [74], children reported increased social anxiety (*d* = 0.07), although the result was not statistically significant (*p* = 0.220). This may be due to the small sample size compared to other studies that included child and adolescent samples. In Zhu et al. [43], social anxiety increased in children from pre-pandemic to post-pandemic (*d* = 0.06), although it is unknown whether statistical significance was achieved. The most influential results were ones produced by longitudinal studies with large sample sizes: Zhu et al. [43] and Charmaraman et al. [49], which were almost identical in sample characteristics, although the former was a study from China, and the latter, the USA. As the results from Zhu et al. are unlikely to be clinically meaningful—whilst those Charmaraman et al.’s results may be quantified as such—there are likely cultural and geographical effects that might determine the extent to which social anxiety has been heightened in the child and adolescent populations.

These results indicate that social anxiety has been exacerbated in adults due to the COVID-19 pandemic; however, the effect sizes are likely small, and there is mixed evidence that adolescents and children have experienced heightened social anxiety. Whilst a range of effect sizes was reported, longitudinal studies tended to report smaller effect sizes, which are more likely to represent the true value of the impact of the pandemic on social anxiety. In the context of global populations, we believe that even small effect sizes are meaningful, as whilst the consequential behavioural and mental processes may not be significantly affected, the cumulative effect of this small change across the entire populace may be insurmountable. Additionally, the observed increased social anxiety may not necessarily be diffuse over the entire population: it is likely that certain individuals are more predisposed to social anxiety than others, meaning a greater proportion of people have had a marked increase; however, a small effect size may not be representative of this, as some people have reported no change or even a decrease in scores. Another important consideration is that the studies only included samples from six countries: China, Turkey, the USA, Germany, Australia, and the UK. It is highly likely that there is variation in the degree to which the pandemic has impacted the prevalence of social anxiety across communities. Other forms of anxiety, such as health-related anxiety, have been found to be exacerbated in regions of more significant infectious outbreaks [75]. In sum, the impact the coronavirus pandemic has had on social behaviours, and relations may directly contribute to increased social anxiety. This is not unexpected, as various mental health models emphasize the contribution of socio-environmental factors to mental health, including the diathesis–stress model [76,77] and the biopsychosocial model [78]. Whilst not all stressful circumstances, real or imagined, may lead to the occurrence of psychopathology, psychosocial stress factors and personal life events can have robust effects on the development of anxiety-related outcomes [79]. 

### 3.4. Social Anxiety in Specific Populations due to the COVID-19 Pandemic

#### 3.4.1. Gender Effects

During the pandemic, women have generally reported elevated social anxiety levels, although studies indicate various effect sizes (Range of Cohen’s *d*: −0.02 to 0.52). Preliminary evidence from longitudinal studies indicates that women have shown an increased risk of social anxiety throughout the duration of the COVID-19 pandemic. In one such study, Juvonen et al. [59] found that women had heightened social anxiety from pre-pandemic to post-pandemic than men (*d* = 0.17). In another longitudinal study, Charmaraman et al. [49] also reported that scores on social anxiety measures compared from pre-pandemic to post-pandemic were higher for women than for men (*d* = 0.52). These results are consistent with data from cross-sectional studies. For instance, Ju et al. [58] found that women scored higher than men on social anxiety (*d* = 0.43). In Zhu et al. [43], female students reported significantly higher social anxiety compared to male students (*p* < 0.001). Finally, Falco et al. [52] found that women had increased social anxiety during a lockdown, with a small effect size (Hedge’s *g* = 0.33, *p* = 0.001).

Although in the minority, there have been some studies that do not show significant gender differences. Notably, three cross-sectional studies reported higher, but statistically non-significant, social anxiety scores for women compared to men (*d* = 0.29, *d* = 0.04, and *d* = 0.08) [57,62,63]. Moreover, Lim et al. [64] found that gender was not a predictor of social anxiety at the onset (*d* = 0.06) or throughout the pandemic (*β* = −0.02, *p* < 0.003). However, in this study, no data were collected during the pre-pandemic period, so inferences are limited.

Generally, women scored higher for social anxiety in the included studies. This may simply represent the higher prevalence rates of social anxiety in women that existed prior to the pandemic and have been evident across many countries [80,81]. However, the evidence from the longitudinal data suggests that the COVID-19 pandemic has specifically produced heightened social anxiety responses in women. The results from the longitudinal studies with large sample sizes indicate an effect size that could be considered clinically significant by certain thresholds [82]. Many studies have found similar results for other mental health issues [83], such as mental distress [84], generalized anxiety [85], eating-disorder hospital admissions [86], and concern and fear regarding COVID-19 [29]. Reasons are likely multifactorial, including movement restrictions that disproportionately affect occupations with higher rates of women workers [87,88]. Further research is needed, therefore, to determine factors that might influence gender differences. 

#### 3.4.2. Financial Stress

Financial stress is associated with post-pandemic social anxiety, and income has been found to have a negative relationship with social anxiety. In Ju et al. [58], social anxiety was higher in a no-fixed income category compared to income-earning categories—although *p* values were predominantly statistically non-significant. In Itani et al. [57], monthly family income was negatively associated with severe social anxiety in adolescents (Odds Ratio [*OR*] = 0.32, 95% CI: 0.14 to 0.76). In Lim et al. [64], having lower than average wealth predicted social anxiety at the start of the pandemic (*β* = 0.12, *p* < 0.001), along with being unemployed (*β* = 0.14, *p* < 0.001). Finally, in a longitudinal study, Juvonen et al. [59] found that financial stress was predictive of social anxiety during the pandemic for young adults (*β* = 0.14, *p* < 0.001). Taken together, these findings suggest that individuals with lower financial resources may be a vulnerable subpopulation. This is consistent with research indicating that socioeconomic patterns have affected psychopathology during the pandemic [88] and lockdowns [84]. Individuals with fewer financial resources are exposed to more pandemic-related stressors [89,90,91,92], experience multiple daily life stressors and have fewer coping resources, and are therefore subjected to higher rates of negative mental health outcomes, such as social anxiety, than their counterparts.

### 3.5. The COVID-19 Pandemic’s Effects on Individuals with SAD

Individuals with a diagnosis of SAD have shown an increased need for therapeutic support and a deterioration in their mental health during the pandemic. Bendau et al. [45] measured post-pandemic social anxiety across four time points throughout the pandemic (see Table 1). Individuals with SAD scored higher on depression (PHQ-2; *b* = 0.50, *p* < 0.01), generalized anxiety (GAD-2; *b* = 0.43, *p* < 0.01), and both symptoms together (PHQ-4; *b* = 0.50, *p* < 0.01)—and there was no statistically significant change in such symptoms over time points during the pandemic. Additionally, the authors found no change in disorder-specific anxiety symptoms during the pandemic for participants who identified as having SAD. In Quittkat et al. [70], 27.9% of the participants that identified as having SAD reported an increased need for therapeutic support compared to how they felt before the pandemic. Further, for perceived changes in mental health during COVID-19, 33.72 % reported that they were slightly worsened, and 8.14% reported that they worsened considerably. More specifically, Carlton et al. [48] found that compared to those without SAD, individuals with SAD had higher rates of depression (*t*(83) = 3.74, *p* < 0.05), general anxiety (*t*(83) = 4.80, *p* < 0.001), and stress (*t*(83) = 4.00, *p* < 0.001).

People with SAD have reported significantly exacerbated responses to stress as compared to their non-socially anxious peers. Carlton et al. [48] examined SAD in response to COVID-19 pandemic-related stress. For those with a SAD diagnosis, disengagement coping (i.e., “when I am around other people I act like COVID-19 never happened”) was greater, reaching statistical significance (*d* = 0.69, *p* < 0.05). Disengagement coping is negatively associated with psychological well-being [93], and these results indicate that those with social anxiety respond to the threat of COVID-19 with avoidance, denial, and wishful thinking. Additionally, SAD individuals had higher Stress Involuntary Engagement (*d* = 1.18, *p* < 0.01)—for instance, rumination, intrusive thoughts, physiological and emotional arousal, and involuntary action—and scored higher (*d* = 1.28, *p* < 0.001) on Stress Involuntary Disengagement (e.g., emotional numbing, cognitive interference, inaction, and escape). Carlton et al. [48] also assessed the relationship between SAD and the Fear of Illness and Virus Evaluation (FIVE). Those with SAD scored higher compared to non-SAD individuals (*d* = 1.17, *p* < 0.001) for fears relating to contamination and illness (e.g., “I am afraid I will have to go to the hospital because of a bad Illness or virus). Moreover, SAD individuals scored higher (*d* = 1.02, *p* < 0.001) for fears associated with social distancing (e.g., “I am afraid I will lose my friends because of a bad illness or virus”) and higher (*d* = 1.19, *p* < 0.01) for how much these fears impacted the participant’s life (e.g., “being afraid of an illness or virus has caused me to feel experience strong emotions”). Consistent with these results, Falco et al. [52], using a DSM-5-based self-report questionnaire, conducted a path mediation and found intrusive stress (e.g., repeated thoughts about the stressful event) to be related to social anxiety (*β* = 0.21, 99% CI > 0.0). Additionally, hyperarousal stress, such as anger and irritability, difficulty concentrating and hypervigilance, was related to social anxiety (*β* = 0.17, 99% CI > 0.0).

Individuals with SAD who had previously sought psychotherapy before the onset of the pandemic appear to have coped well with lockdowns and the pandemic. In Samantaray et al. [71], medical students with SAD had completed Cognitive-Behavioural Therapy or Psychoeducational-supportive therapy. Those who had completed CBT did not have statistically significant changes in social anxiety during a lockdown. However, those with mental health comorbidity (e.g., Major Depressive Disorder) had significantly higher social anxiety scores during the lockdown (*p* < 0.05).

For many individuals with SAD, mental health has deteriorated during the COVID-19 pandemic. However, this is common across mental health conditions because they predispose individuals with clinical vulnerability to negative mental health outcomes [94,95]. Those with SAD tend to overestimate threats, a transdiagnostic marker of anxiety disorders [96], and this can produce the elevated stress responses and depressive and anxiety symptoms that have arisen within the context of the COVID-19 pandemic. Lockdowns appear to be particularly difficult for those with SAD who have a co-morbid condition [71]. Naturally, clinicians are advised to continue treatment of individuals with SAD and to monitor negative mental health symptoms that may be from increased pandemic stressors. Unfortunately, no longitudinal studies have directly assessed whether social anxiety symptoms were affected by the COVID-19 pandemic in individuals with clinical diagnoses of SAD.

### 3.6. Risk and Protective Factors Influencing Social Anxiety Levels during the COVID-19 Pandemic

#### 3.6.1. Contracting COVID-19

Receiving a positive SARS-CoV-2 virus test result has become a risk factor for social anxiety, and internalized stigma is a possible mediator in the relationship between contracting COVID-19 and social anxiety. This is congruent with previous research indicating that internalized shame contributes to social anxiety outcomes over and above depression [97]. In Czorniej et al. [50], individuals who had contracted the COVID-19 virus had significantly higher scores on social anxiety (*p* < 0.001). In a study by Ju et al. [58], receiving a positive SARS-CoV-2 nucleic acid test result was associated with increased social anxiety six months later (*β* = 0.19, *p* = 0.008). A path mediation mode indicated that low social support, perceived stigma, and negative treatment from others due to a COVID-19 diagnosis predicted internalized stigma of being infected by the virus, and internalized stigma predicted social anxiety. That a positive SARS-CoV-2 virus result is associated with social anxiety is consistent with other recent studies showing individuals with severe acute COVID-19 illness to have increased depressive and anxiety symptoms [98]. We speculate this relationship may weaken over time as receiving a positive SARS-CoV-2 virus result naturally becomes more common and less stigmatized.

Along with contracting COVID-19, concern about contracting COVID-19 may also be a risk factor for social anxiety. Samantaray et al. [71] found that fear of COVID-19 accounted for 49.6% variability in social anxiety (SPIN) during lockdowns and was correlated with post-pandemic social anxiety (*r* = 0.60, *p* < 0.01). Additionally, Blasco-Belled et al. [46] found that social anxiety correlated significantly with a COVID-19 threat item. In Falco et al. [52], scores on fear of COVID-19 were large for a high social anxiety group compared to a low social anxiety group, reaching an almost moderate effect size (Hedge’s *g* = 0.47, *p* = 0.003). In Moran [68], fear of COVID-19 had a positive but non-significant correlation with social anxiety (*r* = 0.12, *p* = 0.52), although this was possibly due to the small sample size (*n* = 32). Additionally, for children, Terin et al. [73] discovered that increased social anxiety scores were detected among a group who are worried about being diagnosed with COVID-19 (*t*(197) = 2.73, *p* < 0.007).

There have been some contrary findings. For instance, Hawes et al. [54] found that the concerns of COVID-19 infection were not significantly related to social anxiety in multivariable linear regression. Nevertheless, generally, the results indicate that the mere threat of infection can exacerbate social anxiety and other anxiety disorders [99,100]. However, within these studies, inferences of directionality are limited: individuals with higher social anxiety may have an increased threat response, or individuals who are sensitive to anxiety-provoking events may report higher social anxiety.

#### 3.6.2. Socio-Emotional Well-Being and Coping Style

Coping style and pre-pandemic social-emotional well-being have been found to be predictors of social anxiety during the pandemic. In Juvonen et al. [59], pre-pandemic social-emotional well-being predicted post-pandemic social anxiety (*β* = 0.50, *p* < 0.001). In Li [62], psychological capital—encompassing hope, optimism, self-efficacy, and resilience—was negatively correlated with negative coping (*r* = −0.19), and negative coping was positively correlated with social anxiety (*r* = 0.43), with a relative mediating effect of 6.92%. Conversely, psychological capital had a positive correlation with positive coping (*r* = 0.38), and positive coping was negatively correlated with social anxiety (*r* = −0.16), with a relative mediating effect of 22.35% between psychological capital, positive coping, and social anxiety. In Ma [65], resilience, a facet of psychological capital, had a significant relationship with social anxiety (*r* = −0.66, *p* < 0.001). Fawwaz et al. [53] collected data during a lockdown, finding that interpersonal mattering, the inclination to perceive oneself as important to others, had a non-significant relationship with social anxiety (*β* = −0.04, *p* < 0.618). However, they discovered that societal mattering, the feeling that one makes a difference in society, had a significant relationship (*β* = −0.41, *p* < 0.001).

In sum, those with lower levels of psychological capital and a negative coping style are likely to be at increased risk of developing social anxiety symptoms during the pandemic. In contrast, socio-emotional well-being, positive coping style, and societal mattering may be protective factors against social anxiety. Although studies have only included measures that are broad in conceptual scope, the findings were consistent with previous research. Avoidance coping (i.e., relieving negative emotions to stressful events through evasion) is a risk factor for anxiety [101], and socio-emotional well-being and adaptive coping (i.e., positive attitudes to cope with stressful events) is a negative predictor of social anxiety during stressful events [102,103].

#### 3.6.3. Social Networks and Friendships

Friendships, particularly close friendships, have been a protective factor against social anxiety during the pandemic. In Thompson et al. [42], an individual’s pre-pandemic social network—measuring both the amount and quality of friendships—was negatively associated with social anxiety during the pandemic (*r* = −0.31, *p* < 0.01). In Juvonen et al. [59], increases in the number of friendships during COVID-19 (*β* = −0.08, *p* < 0.001) and in friendship quality (*β* = −0.05, *p* < 0.05) were found to be negatively related to social anxiety during continued public health restrictions. In Itani et al. [57], increased frequency of friendship interactions negatively predicted severe social anxiety (*OR* = 0.38, 95% CI: 0.17 to 0.88). Lastly, Moran [68] found that perceived social support was a significant negative predictor of social anxiety (*β* = −0.63, *p* < 0.05) in a sample of domestic violence survivors. Taken together, the research suggests that social networks have been a determinant of social anxiety during the pandemic, and this is consistent with research showing that social support promotes wellness [101], mitigates the effect of life stressors [102,104], and is negatively associated with social anxiety [105]. One explanation for this, according to the stress-buffering model [106], is that social support may discourage the perception of situations as threatening and bolster the perception that greater resources are available [107].

#### 3.6.4. Student/Work Mode

There is little evidence that remote work or study has influenced social anxiety during the pandemic. In Juvonen et al. [59], remote work and study were non-significantly associated with social anxiety during the pandemic. Similarly, Eskiyurt & Akaca [51] reported no significant relationship between social anxiety scores and feeling more anxious in either virtual or classroom environments. Although a study by Liang et al. [63] found that online teaching methods were associated with higher social anxiety, no post hoc analyses were conducted. 

### 3.7. Responses to the COVID-19 Pandemic due to Social Anxiety

#### 3.7.1. Educational Environments

There is some evidence that educational environments that disallow exposure to feared social situations (e.g., due to social distancing) may contribute to the maintenance of social anxiety. Using longitudinal data, Arad et al. [44] assessed levels of social anxiety at the beginning and end of the academic year for socially anxious university students during the pandemic. Data were compared to a group from previous years (i.e., individuals that had not experienced the pandemic). The group that was not subjected to the pandemic showed a significant (*t*(43) = 7.4, *p* < 0.001) decrease in social anxiety at the end of the school term, with a large effect size of *d* = 1.12. Conversely, the students who experienced social distancing during the pandemic showed a non-significant reduction in their levels of social anxiety (*d* = −0.06, *p* = 0.066). A comparison of the two cohorts showed that the pandemic group was significantly higher in social anxiety than the pre-pandemic group (*t*(97) = −5.08, *p* < 0.001) with a large effect size (*d* = 1.03). Two other studies have explored social avoidance behaviours within virtual environments. In Eskiyurt & Akaca [51], individuals with high social anxiety recorded their reasons for their preference for virtual environments, and these indicated the increased opportunities afforded in virtual environments to self-conceal (e.g., “Since I couldn’t open the camera during online classes, I felt more comfortable while talking,” and “I felt more comfortable since no one saw me”).

Other studies have found significant differences in social anxiety according to the communication mode of students. Liang et al. [63] reported that students who communicated primarily with character communication (e.g., email), as opposed to video, face-to-face, or telephone, had the highest social anxiety scores. As socially anxious people are more likely to see themselves from an observer’s perspective [108], personal modes of communication (e.g., face-to-face) may be more socially threatening for them. In contrast, character communication may allow for social contact without fear of immediate disapproval and assist the socially anxious person with achieving self-presentational goals.

Overall, the findings indicate that virtual environments may facilitate self-concealment and avoidance of situations that require closer interpersonal distances. Individuals with social anxiety typically avoid opportunities for social interaction [109], which may otherwise provide corrective information, thereby maintaining their social anxiety symptoms [108,110]. With the wide use of virtual work environments and online learning during the pandemic, it will be beneficial for occupational and educational systems to adapt their services to users, allowing them more engagement and affiliative responsibility. 

#### 3.7.2. COVID-19 Knowledge

There is mixed evidence to suggest that knowledge about COVID-19 is directly related to social anxiety, and positive and negative effects may mediate the relationship between social anxiety and the degree of life satisfaction, which is typically reduced for socially anxious people [111]. In Blasco-Belled et al. [46], social anxiety was found to negatively influence life satisfaction directly and indirectly through the degree of knowledge relating to COVID-19. Specifically, there were negative direct effects between social anxiety and life satisfaction (*β* = −0.56, *p* < 0.001) and between social anxiety and COVID-19 (*β* = −0.36, *p* < 0.05). However, the indirect effect between COVID-19 knowledge and life satisfaction was positive (*β* = 0.15, *p* < 0.001). The authors argued that individuals with social anxiety tend to dwell on negative thoughts, promoting the avoidance of stressful situations and reminders of them (e.g., COVID-19). Another finding was that positive and negative affectivity—how much individuals experience positive and negative emotions—were significant moderators of the relationship between social anxiety and life satisfaction in the context of COVID-19. Thus, the indirect effect of social anxiety on life satisfaction through COVID-19 knowledge decreased with a higher positive effect, whereas the indirect effect increased with a higher negative effect. However, another study by Itani et al. [57] found no association between the participants’ understanding of COVID-19 and severe social anxiety. This is likely due target population and comparison group of the study. We would not expect the reason that individuals who quantified as having severe social anxiety was due simply because of a greater degree of knowledge about COVID-19.

#### 3.7.3. Social Media

Research has established that exposure to COVID-19-focused news through social media is generally associated with negative mental health symptoms [112,113,114]. Whilst previous literature generally reports that social media usage is directly associated with social anxiety [115], directionality cannot be assumed. The evidence is mixed, however, as to whether social media usage during the pandemic is positively correlated with social anxiety. There is also some evidence that exposure to COVID-19 information is a mediating variable in this association. In Itani et al. [57], social media usage was positively correlated with severe social anxiety (*OR* = 2.65, 95% CI: 1.21 to 5.80). Likewise, Pang [69] found that compulsive WeChat use (a social media platform) was positively correlated with social anxiety, although this correlation was non-significant when entered into an SEM model. Instead, social media fatigue—fatigue and boredom arising from participation in social media activities—which is often attributed to information overload [116], became a significant mediator between social media usage and social anxiety. Specifically, exposure to COVID-19-related information contributed to social media fatigue (*r* = 0.58, *p* < 0.001), and social media fatigue was related to social anxiety (*r* = 0.69, *p* < 0.001). As such, problematic social media use may heighten social anxiety through social media fatigue.

#### 3.7.4. COVID-Related Anxiety and Worry

Pre-pandemic social anxiety has been associated with COVID-19-related anxiety in adults but not in adolescents. Ho and Moscovitch [55] retrospectively assessed social anxiety that accounted for 36% of the variance on the Coronavirus Anxiety Scale, a measure of dysfunctional anxiety associated with the coronavirus pandemic. Likewise, Buckner et al. [47] found that social anxiety was related to COVID-19-related worry (e.g., “I am worried I will lose friends due to social distancing”) after controlling for pre-pandemic anxiety and depression (*β* = 0.34, *p* = 0.001). In Bendau et al. [45], individuals who self-identified as having SAD had higher COVID-19-related fear (*b* = 0.22, *p* < 0.001). However, these results cannot be readily extrapolated to adolescent populations. In Morales et al. [67], adolescents with higher pre-pandemic social anxiety—after accounting for the effects of generalized anxiety—reported fewer COVID-19-related worries during a lockdown and during the pandemic.

Overall, the findings from studies with adult samples point to positive associations between social anxiety and COVID-19-related anxiety and theoretically associated constructs. This suggests that people with subsyndromal social anxiety, and those with SAD, overestimate the potential threat of COVID-19 due to the interactions between clinical vulnerabilities and pandemic stressors [94]. However, the association seems to have been particularly prominent earlier in the pandemic. To clarify these findings, future research is required that considers age effects, generalized anxiety as a mediator, and which of the specific facets of COVID-19-related anxiety are most affected.

#### 3.7.5. Affiliative Responses

Investigations of the associations between social anxiety and affiliative responses during the pandemic have produced mixed results. Huang et al. [56] found that social anxiety was positively correlated with interpersonal distancing (*r* = 0.10, *p* = 0.04). Additionally, Krämer et al. [60] found that social anxiety was not associated with personal contact (e.g., face-to-face interactions) during a lockdown, although it was negatively associated ($\hat{y}$ = −0.02, *p* = 0.012) with indirect contact, such as text messages. In contrast, other studies have found social anxiety to be associated with increased affiliative responses. In Buckner et al. [47], higher pre-lockdown social anxiety was related to keeping in contact with family and friends (*r* = 0.22, *p* < 0.05). In addition, Ho and Moscovitch [55] retrospectively assessed social anxiety, which was a positive predictor of affiliative frequency, explaining 14.7% of the variance (*R*^2^ = 0.15, *p* < 0.001). However, this relationship was moderated by the degree of functional impairment and the experience of COVID-related stressors. Socially anxious individuals with the greatest functional impairment and those experiencing the greatest COVID-19-related stressors reportedly engaged in the most affiliative behaviours. 

These mixed findings may be due to limitations in the measures that have been used to assess social anxiety. For instance, retrospectively assessed self-reports of social anxiety may be confounded by recall bias, which may distort judgments depending on the person’s current state. Another limitation is the exclusion of potential mediators or moderators of the measures used (e.g., extroversion; self-esteem; quality of relationships). For example, the socially anxious individuals, who have been most impaired due to the pandemic and who report higher affiliative frequency, may be attempting to obtain social support to help moderate the effects of pandemic stressors.

#### 3.7.6. Loneliness and Friendship

The research suggests that social anxiety has been associated with increased loneliness during the pandemic. In Ho and Moscovitch [55], retrospective social anxiety accounted for 26% of the scores on loneliness (*p* < 0 .001). Thompson et al. [42] found that social anxiety symptoms were related to loneliness, pre-pandemic (*r* = 0.53, *p* < 0.01) and post-pandemic (*r* = 0.46, *p* < 0.01), and decreased social networks during the pandemic (*r* = −0.31, *p* < 0.01). 

There is also evidence to indicate that, whilst social anxiety is associated with loneliness, the easing of social restrictions may alleviate this effect. Lim et al. [64] reported that although social anxiety was positively related to loneliness at the start of the pandemic (*β* = 0.60, *p* < 0.001), social anxiety negatively predicted loneliness over the next six months as restrictions eased (*β* = −0.54, *p* < 0.001). This finding suggests that loneliness was particularly heightened for socially anxious people during the period of severe social restrictions early in the pandemic. In addition, the results are consistent with previous research, indicating that loneliness and social anxiety predict subsequent changes in each other [117,118], forming a deleterious cycle. In other words, socially anxious individuals often avoid social contact that would reduce loneliness and vice versa.

#### 3.7.7. Depression, Generalized Anxiety, and Stress during Lockdowns

Most of the evidence suggests that, in adult populations, pre-pandemic social anxiety has been a predictor of depression, generalized anxiety, and stress during lockdowns. Thompson et al. [42] retrospectively assessed pre-pandemic social anxiety symptoms, which were positively and significantly associated with depression during the pandemic (Pearson correlations ranging from 0.29 to 0.45; *p* < 0.01). Similarly, in Krämer et al. [60], social anxiety was related to depression and anxiety during a lockdown ($\hat{y}$ = 0.38, *p* < 0.001) and negatively related to life satisfaction ($\hat{y}$ = −0.85, *p* < 0.001). While causality cannot be inferred from these cross-sectional studies, a longitudinal study by Buckner et al. [47] found that pre-pandemic social anxiety predicted lockdown anxiety after controlling for pre-pandemic anxiety and pre-pandemic depression (*β* = 0.25, *p* = 0.002) and lockdown depression (*β* = 0.25, *p* = 0.002). 

The relationships among pre-pandemic social anxiety and depression, anxiety and stress during the pandemic have been different for adolescent populations. For adolescents, lockdowns may, in fact, provide relief from negative mental health outcomes for socially anxious individuals. In a longitudinal study conducted by Morales et al. [67], adolescents high in social anxiety displayed less generalized anxiety and perceived stress during lockdowns. By the time social restrictions were removed, generalized anxiety and perceived stress did not vary between adolescents with varying pre-pandemic social anxiety. Taken together, the data suggest that whereas lockdowns may exacerbate a variety of negative mental health outcomes for socially anxious adults, for adolescents, they may provide some respite.

#### 3.7.8. Mental Health Outcomes during Easing of Restrictions

It has been suggested that socially anxious individuals might experience relief from negative mental health outcomes during social restrictions only to face acute destabilisation when re-socialising (e.g., when attendance at work and school settings are required). The strongest evidence for this possibility comes from Lim et al. [64], a high-quality study that coded for the social restriction severity of each participant across three time points. Easing of restrictions was found to be associated with increases in social anxiety (*β* = 0.07, *p* = 0.01). Additionally, those high on social anxiety at the first time point, when restrictions were generally stricter, displayed increases in social anxiety at a faster rate as restrictions eased (*β* = 1.68, *p* < 0.001). In other words, social anxiety symptoms appeared to be exacerbated as restrictions eased for those who were socially anxious in the first place. Another study by McLeish et al. [66] found that college students had increased social interaction anxiety when returning to a higher frequency of social encounters after a period of social isolation. In this study, data relating to social interaction anxiety were collected from college students across three time points after the onset of the pandemic. Changes in scores over the course of the pandemic’s first year were non-significant; however, social interaction anxiety was significantly higher than at the onset of the pandemic at the last data collection time point when public health measures were relaxed. 

The association between mental health outcomes and the easing of restrictions for socially anxious people may also be influenced by the frequency of their social contact. Krämer et al. [60] found that, for socially anxious people who engaged in a higher number of social interactions, depression and anxiety increased as restrictions eased. More precisely, scores on depression and anxiety (PHQ-4) were found to increase with a higher frequency of social contact among people with higher social anxiety. This applied regardless of whether the nature of the social contact was personal contact ($\hat{y}$ = 0.08, *p* = 0.044) or indirect contact ($\hat{y}$ = 0.014, *p* = 0.048).

Not all studies have shown an increase in symptomatology with the easing of restrictions. Bendau et al. [45] found that individuals who self-reported a SAD diagnosis reported that their social anxiety symptoms were at their highest during a lockdown. However, their symptoms (i.e., social anxiety, depression, and anxiety) were slightly improved over four time periods with eased social restrictions—although these changes did not reach statistical significance. The heterogeneity observed between Bendau et al.’s results may be due to two reasons. Firstly, some regions may have varied in their control of COVID-19 and results; therefore, the results from Bendau et al. may be obfuscated as there was no measure to determine the degree of social restrictions the participant was experiencing during data collection. As previously highlighted, the effects of the pandemic likely impact the prevalence of social anxiety in communities at a differential rate due to a range of factors, including the degree of governmental mandates, the expansion of COVID-19 cases, and cultural attitudes. Another reason for the differential result is that the differences between these findings and that of Lim et al. [64] and McLeish et al. [66] is that Bendau et al. utilised a clinical sample. Thus, for those with SAD, social anxiety may not be readily alleviated with the easing of restrictions but may imply a return to pre-pandemic levels.

#### 3.7.9. Influence of Pre-Lockdown Social Anxiety on Self Care during Lockdown

Social anxiety has been found to be associated with a variety of problematic behaviours related to self-care during lockdowns. Buckner et al. [47] studied the relationship between social anxiety and general health behaviours during a lockdown. The study found that social anxiety was associated with trouble keeping a daily routine (*r* = 0.29, *p* < 0.01), reduced daily hygiene (*r* = 0.26, *p* < 0.01), exercising less (*r* = 0.37, *p* < 0.01), poor sleep (*r* = 0.30, *p* < 0.01), unhealthy eating (*r* = 0.25, *p* < 0.01), lack of work motivation (*r* = 0.22, *p* < 0.05), and higher fatigue (*r* = 0.37, *p* < 0.01). Whilst these associations suggest that those with high social anxiety may be particularly vulnerable during lockdowns, the researchers did not examine the effects of possible confounding or mediating variables on these correlations.

## 4. Limitations of Existing Research and Directions for Future Research

The current findings need to be considered in light of their limitations. Of the thirty-three studies included in this research, twenty-two were cross-sectional. This means that causal inferences of the relationships found between social anxiety and features of the COVID-19 pandemic cannot be confirmed in these studies. Additionally, as most studies did not involve cross-cultural comparisons, the findings may not be generalizable across the global population. Synthesis across countries is further restricted because the form of restrictions and the cultural attitudes towards such restrictions has varied across countries and over time within countries during the pandemic. Further, the pandemic has disproportionately impacted the countries in which the studies’ samples were located. The prevalence, and psychological sequelae, of social anxiety symptoms, may be related to this—as has been the case with generalized anxiety [119]. Within countries, localities varied in their response to the pandemic, and the large majority of studies did not code the variation in which participants were affected by governmental mandates. The collection of cross-cultural and longitudinal data in future research will improve the generalizability of findings and clarify the implied causal relationships over the course of the pandemic that have been identified in cross-sectional research. 

Other limitations of the existing research are related to the measures utilized in the included studies. A major limitation of many studies is the failure to measure potentially confounding or mediating variables. In the cross-sectional studies, confounds, such as Socio-Economic Status, were rarely controlled for, and consequently, the associations may be distorted. Moreover, several factors can magnify social anxiety symptoms. Examples include generalized anxiety, loneliness, substance use, and depression. A clear need in future research is to incorporate measures that reflect the influence of such confounding and mediational variables over time.

The research of clinical samples has also been limited by a general absence of clinical screening of participants. Of the four studies of individuals with SAD, only two [48,71] implemented a clinical screen instrument as part of their selection procedure. The remaining two studies [45,70] relied on the participant’s self-report of a diagnosis of SAD, which is only a crude indication of clinical status. In future work, formal clinical diagnostic assessments via structured interview assessments will enhance the veracity of the findings. 

Another limitation is that various subpopulations may have experienced exacerbated social anxiety due to the pandemic; however, these may not have been adequately assessed by the included studies. For instance, results indicate that healthcare professionals are subject to heightened anxiety, stress, and sleep quality [33,120]. None of the included studies included data to make inferences about the prevalence of social anxiety in healthcare professionals, and this may be true for other subpopulations.

Study quality assessment was undertaken by both authors; however, this was not conducted independently. Instead, the study quality was discussed, and scores for the QualSyst tool were agreed upon by the authors. Due to the range of study designs and methodologies, we found this process beneficial; however, it is a limitation of the review.

Publication and language bias may pose another threat to the conclusions drawn in the current systematic review if the included studies are not a representative sample of the available evidence. Journals are less likely to publish non-English publications, resulting in systematic reviews being subject to language bias. Additionally, published studies tend to be positively biased as studies reporting non-significant results or weak effect sizes are less likely to be published. We attempted to mitigate this by thoroughly searching the grey literature; however, it is impossible to ascertain whether any studies were missed during the search process. Whilst this is a limitation of all reviews, none of the included studies in the current review were identified by the gray literature search, indicating a higher likelihood of publication bias.

## 5. Clinical Implications

The higher incidence and exacerbation of social anxiety due to the pandemic underscores the need for psychological interventions that can address problematic social anxiety in the post-COVID-19 recovery period. Mental health clinicians are advised to consider people that may be at a higher risk of developing social anxiety, for instance, women and low-income earners. Cognitive behaviour therapy (CBT) appears to be a prominent form of treatment whose use could be expanded on in post-COVID recovery. CBT is well established as the most effective treatment of social anxiety disorder currently available [121], and people treated with CBT for SAD have been found to deal more effectively with lockdowns [71]. The Telehealth format of psychotherapy for SAD also indicates efficacy, despite difficulties in finding opportunities for exposure to feared situations (for adapting exposure therapy to the COVID-19 pandemic, see Khan et al. [122], Peros et al. [123], and Molino et al. [124]). Moreover, as social anxiety commonly co-occurs with depression [125,126] and other conditions such as anxiety disorders and substance abuse [18], it will be important to provide treatments that are effective across comorbid conditions. The use of CBT for SAD is further reinforced by findings that this therapy is efficacious in addressing comorbid conditions simultaneously [121,127]. Clinicians may also benefit from evidence of physical and psychological interventions that may alleviate negative mental health outcomes associated with the COVID-19 pandemic, including yoga [128], mindfulness practice [129,130], and progressive muscle relaxation [131]. 

Community-based interventions and specific mental health promotion strategies may help to prevent dysfunction and to assist well-being across the general population. Promoting social connectedness are likely to be beneficial, both as a protective factor for social anxiety and as a mitigator of the other mental health symptomatology socially anxious people may experience, including loneliness, anxiety, and depression. Reducing news consumption may be a point of focus as an excessive amount of screen time absorbing negative news has been shown to result in adverse mental health symptoms [112,113,114], including social anxiety [115]. As avoidance behaviours are a maintaining factor in social anxiety [132], it will be beneficial for educational and occupational settings to reduce opportunities for people to conceal themselves and engage in specific avoidance strategies on virtual or online platforms. Ensuring that environments remain interactive and prioritising personal modes of communication will help reduce the incidence of social anxiety. Interactive forms of communication would also help produce corrective social experiences for individuals with social anxiety and help them develop a greater tolerance for social fear.

## 6. Conclusions

The current systematic review provides a starting point for understanding how the prevalence and extent of social anxiety and its psychological sequelae have been affected by the COVID-19 pandemic. Overall, the current review suggests that social anxiety is likely to be elevated across the general population and especially in women and low-income earners. However, there is mixed evidence that adolescents have experienced heightened social anxiety, and heterogeneity in the results is likely due to geographic factors. Additionally, for adolescents with high social anxiety prior to the onset of the pandemic, lockdowns may provide some respite, whilst evidence suggests this is not the case for adults. Other factors that are likely to contribute to social anxiety include a positive SARS-CoV-2 virus test result, maladaptive coping styles, socio-emotional well-being, and poor support networks. Pre-pandemic social anxiety also appears to influence a variety of affective, behavioural, and cognitive responses to the COVID-19 environment, and findings indicate that adolescents and children vary in their response to the pandemic. In sum, the COVID-19 pandemic has impacted the prevalence of social anxiety and numerous associated negative mental health outcomes. 

## Figures and Tables

**Table 1:** Characteristics of Included Studies.

Study	Country	Sample Size	Sample Characteristics (SD in Parentheses)	Population Type	Design and Date of Data Collection (dd/mm/yyyy)	Relevant Measures	Qualsyst Score
Arad et al. [44]	Israel	*n* = 99	Mean age (treatment): 22.62 (2.36) Mean age (control): 21.57 (1.90) Gender: 85% female	University students (>50 LSAS)	Natural experiment T1: September 2019 to December 2019 T2: January 2020 to April 2020	LSAS-SR, PHQ-9	0.95 (strong)
Bendau et al. [45]	Germany	*n* = 307	Mean age: 39.64 (11.7) Gender: 71.2% female	Clinical (self-diagnosed)	Longitudinal T1: 27 March 2020 to 6 April 2020 T2: 24 April 2020 to 4 May 2020 T3: 15 May 2020 to 25 May 2020 T4: 5 June 2020 to 15 June 2020	C-19-A, PHQ-4	0.95 (strong)
Blasco-Belled et al. [46]	Spain	*n* = 541	Mean age: 38.82 (15.97) Gender: 65.8% female	Community	Cross-sectional Data collected from 12 March 2020 to 15 March 2020	LSAS-SR, PWI, SPANE	0.91 (strong)
Buckner et al. [47]	USA	*n* = 120	Mean age: 19.8 (1.6) Gender: unspecified	Community	Longitudinal T1: 16 February 2020 to 13 March 2020 T2: 13 April 2020 to 15 May 2020	DASS-21, SIAS, Worry Index	0.91 (strong)
Carlton et al. [48]	USA	*n* = 84	Mean age: 19.5 (1.47) Gender: 73.8% female	Clinical	Repeated cross-sectional T1: January 2021 to March 2021 T2: 1 month after T1	ADIS-5, DASS-21, FIVE, RSQ, SAFE	0.82 (strong)
Charmaraman et al. [49]	USA	*n* = 586	Mean age: 12.53 (1.18) Gender: 53% female	Community	Longitudinal T1: September 2019 to November 2019 T2: October 2020 to December 2020	CESDR-10, COVID-19-Related Grief Scale, PRIUSS, SAS-A	0.95 (strong)
Czorniej et al. [50]	Poland	*n* = 255	Mean age: 24.30 (1.69) Gender: 53.7% female	Healthcare Students	Cross-sectional Data collected from May 2021 to May 2022	LSAS, STAI	0.85 (strong)
Eskiyurt & Akaca [51]	Turkey	*n* = 670	Mean age: 20.77 (2.77) Gender: 82% female	Community	Cross-sectional Data collected from February 2020 to February 2021	B-FNE, LSAS	0.91 (strong)
Falco et al. [52]	Spain	*n* = 439	Mean age: 36.64 (13.37) Gender: 73.1% female	University Community	Cross-sectional Data collected from March 2020 to May 2020	ESTAD Anxiety and depression disorders symptoms scale, F-COVID-19, Impact of event scale-revised	0.86 (strong)
Fawwaz et al. [53]	Malaysia	*n* = 158	Mean age: 21.77 (1.54) Gender: 56.3% female	Community	Cross-sectional Data collected from January 2021 to February 2021	MTOQ, UMS, SPIN	0.73 (good)
Hawes et al. [54]	USA	*n* = 451	Mean age: 17.49 (1.42) Gender: 65.4% female	Community	Longitudinal T1: Pre-pandemic (unspecified) T2: 27 March 2020 to 15 May 2020	CDI, SCARED	0.91 (strong)
Ho & Moscovitch [55]	USA	*n* = 488	Median age: 25–39 (N/A) Gender: 48% female	Community	Cross-sectional Data collected from May 2020	BFNE, CAS, CSS, SDS^a^, SIAS, TILS	0.86 (strong)
Huang et al. [56]	China	*n* = 501	Mean age: 24.31 (7.83) Gender: 63.9% female	Community	Cross-sectional Data collected from March 2020	IPCS, LSAS-SR	0.91 (strong)
Itani et al. [57]	Lebanon	*n* = 178	Median age: 16 years old Gender: 59.0% female	Community	Cross-sectional Data collected from August 2020 to September 2020	LSAS-CA	0.73 (good)
Ju et al. [58]	China	*n* = 199	Mean age: 42.72 (17.53) Gender: 53.3% female	Discharged COVID-19 patients	Cross-sectional Data collected from July 2020 to September 2020	Self-Stigma Scale, Self- consciousness Scale	0.77 (good)
Juvonen et al. [59]	USA	*n* = 1557	Mean age: 22.5 (0.75) Gender: 62% female	Community	Longitudinal T1: Pre-pandemic (2017–2019) T2: March 2021 to June 2021	CES-D, GAD-7, SAS-A	0.95 (strong)
Krämer et al. [60]	Germany	*n* = 190	Mean age: 44.2 (14.18) Gender: 47% female	Community	Longitudinal T1: 6 April 2020 T2: 29 April 2020 T3: 20 May 2020 T4: 10 June 2020	BFI-2, PHQ-4, SIAS-6, SOEP, Unified Motive Scale	0.91 (strong)
Langhammer et al. [61]	Germany	*n* = 47	Mean age: 37.3 (10.78) Gender: 60% female	Clinical outpatient	Cross-sectional Data collected from July 2020	BDI-II, HAMA-A, PAS, PHQ-9, SMSP	0.82 (strong)
Li [62]	China	*n* = 600	Median age: 20 (N/A) Gender: 46% female	Community	Cross-sectional Data collected from March 2020	SIAS	0.68 (adequate)
Liang et al. [63]	China	*n* = 3137	Mean age: N/A Gender: 78.58% female	University students	Cross-sectional Data collected from February 2020	SAD, SAS, SDS^b^	0.82 (strong)
Lim et al. [64]	Australia	*n* = 1562	Mean age = 48.8 Gender: 84.2% female	Community	Longitudinal T1: March 2020 T2: 6–8 weeks after T1 T3: 6–8 weeks after T2	Mini-SPIN, PHQ-8, UCLA Loneliness Scale	1.0 (strong)
Ma [65]	USA	*n* = 23	Mean age: 21.3 (N/A) Gender: 78.3% female	Community	Cross-sectional Data collected from September 2020 to December 2020	CD-RISC-10, GAD-7, SM-SAD	0.41 (limited)
McLeish et al. [66]	USA	*n* = 934	Mean age: 20.4 (3.59) Gender: 72.4% female	University students	Repeated cross-sectional T1: March 2020 to May 2020 T2: September 2020 to December 2020 T3: January 2021 to April 20201	OASIS, ODSIS, PSWQ-3, SIAS-6, SPS-6, SSASI	0.73 (good)
Morales et al. [67]	USA	*n* = 164	Mean age: 16.16 (0.61) Gender: N/A	Community	Longitudinal T1: March 2017 to August 2019 T2: April 2020 to May 2020 T3: June 2020 to July 2020 T4: August 2020 to September 2020	SCARED, CASPE, GAD-7, K-SADS, PSS-10	0.95 (strong)
Moran et al. [68]	USA	*n* = 32	Mean age: 27.9 (4.52) Gender: 90.6% female	Domestic Violence Survivors	Cross-sectional Data collected from January 2021 to March 2021	ABI, IDAS, MAC-RF, MSPSS	0.65 (adequate)
Pang [69]	China	*n* = 566	Median age: 21–23 (N/A) Gender: 58.8% female	Community	Cross-sectional Data collected from April 2020		0.82 (strong)
Quittkat et al. [70]	Germany	*n* = 86	Mean age: 33.41 (11.45) Gender: 73% female	Clinical (self-diagnosed)	Cross-sectional Data collected from 2 April 2020 to 6 May 2020	PHQ-9, SIAS, SPS	0.95 (strong)
Samantaray et al. [71]	India	*n* = 65	Mean age: 21.77 (2.67) Gender 53.8% female	Clinical	Cross-sectional Data collection unspecified.	F-COVID-19, SPIN	0.83 (strong)
Tekin [72]	Turkey	*n* = 118	Mean age: 13.2 (2.1) Gender 65% female	Community	Cross-sectional Data collected from March 2021 to April 2021	ARI-P, RCADS-P, PedsQL, Turgay-DSM-IV-S	0.77 (good)
Terin et al. [73]	Turkey	*n* = 199	Mean age: 14.48 (2.15) Gender: 74.6% female	Hospital patients	Cross-sectional Data collected from October 2021 to January 2022	CAS, CASI, RCADS-CV	0.91 (strong)
Thompson et al. [42]	USA	*n* = 204	Mean age: 30.4 (11.2) Gender: 83.2% female	Community	Cross-sectional Data collected from September 2020	LSNS-6, SPS, UCLA Loneliness scale	0.55 (adequate)
Yurteri & Sarigedik [74]	Turkey	*n* = 60	Mean age: 10.22 (N/A) Gender: 46.7% female	Community	Longitudinal T1: Pre-pandemic (unspecified) T2: Post-Pandemic (unspecified)	CDI, SCARED	0.86 (strong)
Zhu et al. [43]	China	*n* = 1393	Mean age: 13.04 (0.86) Gender 53.1% female	Community	Longitudinal T1: September 2019 T2: June 2020	GAD-7, PHQ-9, MSPSS, MDASS, SAS, SCS, STAI, SWLS	0.95 (strong)

Notes: When mean age was not available, median age was included. ABI, Abusive Behavior Inventory; ARI-P, Affective Reactivity Index—Parent-Report; BDI-II, Beck Depressive Inventory; BFI-2, Big Five Inventory-2; BFNE, Brief Fear of Negative Evaluation; C-19-A, COVID-19-Anxiety Questionnaire; CAS, Coronavirus Anxiety Scale; CASI, Anxiety Sensitivity Index for Children; CASPE, COVID-19 Adolescent Symptom & Psychological Experience Questionnaire; CDI, Child Depression Inventory; CES-D, Center for Epidemiologic Studies Depression Scale; CESDR-10, Center for Epidemiologic Studies Depression Scale Revised; CD-RISC-10, Connor-Davidson Resilience Scale 10-item; CSS, Coronavirus Stressor Survey; DASS-21, Depression, Anxiety and Stress Scale—21 Items; F-COVID-19, Fear of COVID-19 scale; FIVE, Fear of Illness and Virus Evaluation; GAD-7, General Anxiety Disorder-7, HAM-A, Hamilton Anxiety Rating Scale; IDAS, Inventory of Depression and Anxiety Symptoms; K-SADS, Kiddie Schedule for Affective Disorders and Schizophrenia; IPCS, Interpersonal Curiosity Scale; LSAS-CA, Liebowitz Social Anxiety Scale for Children and Adolescents; LSAS-SR, Liebowitz Social Anxiety Scale—Self Report; LSNS-6, Lubben Social Network Scale-6; MAC-RF, Multidimensional Assessment of COVID-19-Related Fears; MDASS, Mind-sets of Depression, Anxiety, and Stress; MSPSS, Multidimensional Scale of Perceived Social Support; MTOQ, Mattering to Others Questionnaire; N/A, Not Available; OASIS, Overall Anxiety Severity and Impairment Scale; ODSIS, Overall Depression Severity and Impairment Scale; PAS, Panic and Agoraphobia Scale; PedsQL, Pediatric Quality Of Life Inventory; PHQ-4, Patient Health Questionnaire for Depression and Anxiety; PHQ-9, Patient Health Questionnaire-9; PRIUSS, Problematic Internet Use Scale; PSS-10; Perceived Stress Scale; PSWQ-3, Ultra-Brief Penn State Worry Questionnaire; PWI, Personal Wellbeing Index; RCADS-CV, Revised Child Anxiety and Depression Scale—Child Version; RCADS-P, Revised Children Anxiety And Depression Scales—Parent Form; RSQ, Response to Stress Questionnaire for COVID-19; SAD, Social Avoidance and Distress Scale; SAFE, Subtle Avoidance and Frequency Examination; SAS, Self-Rating Anxiety Scale; SAS, Social Anxiety Scale; SAS-A, Social Anxiety Scales for Adolescents; SCS, Self-Control Scale; SCARED, Screen for Child Anxiety Related Disorders; SD, Standard Devation; SDS^a^, Sheehan Disability Scale; SDS^b^, Self-Rating Depression Scale; SDS^c^, Bogardus Social Distance Scale; SIAS, Social Interaction Anxiety Scale; SIAS-6, Short Form Social Interaction Anxiety Scale; SM-SAD, Severity Measure for Social Anxiety Disorder; SOEP, Socio-Economic Panel; SMSP, Severity Measure for Specific Phobia; SPANE, Scale of Positive and Negative Experience; SPIN, Social Phobia Inventory; SPS, Social Phobia Scale; SPS-6, Social Phobia Scale; SSASI, Short Scale Anxiety Sensitivity Index; STAI, State-Trait Anxiety Inventory; SWLS, Satisfaction With Life Scale; T, Timepoint; TDDS, Three-Domain Disgust Scale; TILS, Three Item Loneliness Scale; UMS, University Mattering Scale.

## Data Availability

Not applicable.

## Appendix A

**Table A1:** PRISMA Checklist.

Section and Topic	Item #	Checklist Item	Location Where Item is Reported
**TITLE**			
Title	1	Identify the report as a systematic review.	Page 1
**ABSTRACT**			
Abstract	2	See the PRISMA 2020 for Abstracts checklist.	Page 1
**INTRODUCTION**			
Rationale	3	Describe the rationale for the review in the context of existing knowledge.	Pages 1–2
Objectives	4	Provide an explicit statement of the objective(s) or question(s) the review addresses.	Page 2
**METHODS**			
Eligibility criteria	5	Specify the inclusion and exclusion criteria for the review and how studies were grouped for the syntheses.	Page 3
Information sources	6	Specify all databases, registers, websites, organisations, reference lists, and other sources searched or consulted to identify studies. Specify the date when each source was last searched or consulted.	Page 3
Search strategy	7	Present the full search strategies for all databases, registers and websites, including any filters and limits used.	Page 3 and Appendix A
Selection process	8	Specify the methods used to decide whether a study met the inclusion criteria of the review, including how many reviewers screened each record and each report retrieved, whether they worked independently, and, if applicable, details of automation tools used in the process.	Page 3
Data collection process	9	Specify the methods used to collect data from reports, including how many reviewers collected data from each report, whether they worked independently, any processes for obtaining or confirming data from study investigators, and, if applicable, details of automation tools used in the process.	Page 4
Data items	10 a	List and define all outcomes for which data were sought. Specify whether all results that were compatible with each outcome domain in each study were sought (e.g., for all measures, time points, analyses), and if not, the methods used to decide which results to collect.	Appendix C
	10 b	List and define all other variables for which data were sought (e.g., participant and intervention characteristics, funding sources). Describe any assumptions made about any missing or unclear information.	Appendix C
Study risk of bias assessment	11	Specify the methods used to assess risk of bias in the included studies, including details of the tool(s) used, how many reviewers assessed each study and whether they worked independently, and, if applicable, details of automation tools used in the process.	Page 4
Effect measures	12	Specify for each outcome the effect measure(s) (e.g., risk ratio, mean difference) used in the synthesis or presentation of results.	Page 4
Synthesis methods	13 a	Describe the processes used to decide which studies were eligible for each synthesis (e.g., tabulating the study intervention characteristics and comparing against the planned groups for each synthesis (item #5)).	Not Applicable
	13 b	Describe any methods required to prepare the data for presentation or synthesis, such as handling of missing summary statistics or data conversions.	Not Applicable
	13 c	Describe any methods used to tabulate or visually display the results of individual studies and syntheses.	Not Applicable
	13 d	Describe any methods used to synthesize results and provide a rationale for the choice(s). If meta-analysis was performed, describe the model(s), method(s) to identify the presence and extent of statistical heterogeneity, and software package(s) used.	Not Applicable
	13 e	Describe any methods used to explore possible causes of heterogeneity among study results (e.g., subgroup analysis, meta-regression).	Not Applicable
	13 f	Describe any sensitivity analyses conducted to assess the robustness of the synthesized results.	Not Applicable
Reporting bias assessment	14	Describe any methods used to assess risk of bias due to missing results in a synthesis (arising from reporting biases).	Page 4
Certainty assessment	15	Describe any methods used to assess certainty (or confidence) in the body of evidence for an outcome.	Page 5–19
**RESULTS**			
Study selection	16 a	Describe the results of the search and selection process, from the number of records identified in the search to the number of studies included in the review, ideally using a flow diagram.	Page 3
	16 b	Cite studies that might appear to meet the inclusion criteria but which were excluded, and explain why they were excluded.	Not Applicable
Study characteristics	17	Cite each included study and present its characteristics.	Table 1
Risk of bias in studies	18	Present assessments of the risk of bias for each included study.	Table 1
Results of individual studies	19	For all outcomes, present, for each study: (a) summary statistics for each group (where appropriate) and (b) an effect estimate and its precision (e.g., confidence/credible interval), ideally using structured tables or plots.	Page 5–19
Results of syntheses	20 a	For each synthesis, briefly summarise the characteristics and risk of bias among contributing studies.	Not Applicable
	20 b	Present results of all statistical syntheses conducted. If meta-analysis was conducted, present for each the summary estimate and its precision (e.g., confidence/credible interval) and measures of statistical heterogeneity. If comparing groups, describe the direction of the effect.	Not Applicable
	20 c	Present results of all investigations of possible causes of heterogeneity among study results.	Not Applicable
	20 d	Present results of all sensitivity analyses conducted to assess the robustness of the synthesized results.	Not Applicable
Reporting biases	21	Present assessments of the risk of bias due to missing results (arising from reporting biases) for each synthesis assessed.	Page 4
Certainty of evidence	22	Present assessments of certainty (or confidence) in the body of evidence for each outcome assessed.	Page 5–19
**DISCUSSION**			
Discussion	23 a	Provide a general interpretation of the results in the context of other evidence.	Page 5–19
	23 b	Discuss any limitations of the evidence included in the review.	Page 19–20
	23 c	Discuss any limitations of the review processes used.	Page 19–20
	23 d	Discuss the implications of the results for practice, policy, and future research.	Page 19–20
**OTHER INFORMATION**			
Registration and protocol	24 a	Provide registration information for the review, including register name and registration number, or state that the review was not registered.	Page 3
	24 b	Indicate where the review protocol can be accessed or state that a protocol was not prepared.	Page 3
	24 c	Describe and explain any amendments to the information provided at registration or in the protocol.	Not Applicable
Support	25	Describe sources of financial or non-financial support for the review and the role of the funders or sponsors in the review.	Page 24
Competing interests	26	Declare any competing interests of review authors.	Page 24
Availability of data, code and other materials	27	Report which of the following are publicly available and where they can be found: template data collection forms; data extracted from included studies; data used for all analyses; analytic code; any other materials used in the review.	Appendix A, Appendix B and Appendix C

## Appendix B

**Table A2:** Search Terms and Strategy.

Database	Search String	Hits	Search Date (dd/mm/yyyy)
EBSCOhost	(“Social Anxiety” OR “Social phob*”) AND (“COVID*” OR “Coronavirus” OR “Pandemic” OR “Lockdown”) (All fields)	69	1 September 2021
		56	1 March 2022
		41	1 August 2022
		11	1 October 2022
EMBASE	(“Social Anxiety” OR “Social phob*”) AND (“COVID*” OR “Coronavirus” OR “Pandemic” OR “Lockdown”) (Title/Abstract)	74	1 September 2021
		27	1 March 2022
		49	1 August 2022
		23	1 October 2022
Cochrane	(“Social Anxiety” OR “Social phob*”) AND (“COVID*” OR “Coronavirus” OR “Pandemic” OR “Lockdown”) (Title or Abstract or Keyword)	4	1 September 2021
		63	1 March 2022
		0	1 August 2022
		2	1 October 2022
Proquest Dissertations and Theses	(“Social Anxiety” OR “Social phob*”) AND (“COVID*” OR “Coronavirus” OR “Pandemic” OR “Lockdown”) (Document title or Abstract)	8	1 September 2021
		0	1 March 2022
		3	1 August 2022
		12	1 October 2022
PsycNET	(“Social Anxiety” OR “Social phob*”) AND (“COVID*” OR “Coronavirus” OR “Pandemic” OR “Lockdown”) (Any Field)	19	1 September 2021
		9	1 March 2022
		11	1 August 2022
		20	1 October 2022
Scopus	(“Social Anxiety” OR “Social phob*”) AND (“COVID*” OR “Coronavirus” OR “Pandemic” OR “Lockdown”) (Title/Abstract/Keywords)	96	1 September 2021
		33	1 March 2022
		68	1 August 2022
		19	1 October 2022
Web of Science	(“Social Anxiety” OR “Social phob*”) AND (“COVID*” OR “Coronavirus” OR “Pandemic” OR “Lockdown”) (Topic)	53	1 September 2021
		46	1 March 2022
		31	1 August 2022
		13	1 October 2022

## Appendix C

**Table A3:** PICO Framework.

PICO Elements	Description
Aim 1: The incidence of social anxiety within the general population because of the COVID-19 pandemic.	
Population/Condition	All individuals affected by the COVID-19 pandemic
Interventions	Onset of the COVID-19 pandemic
Comparator	Not applicable to the general population.Subpopulations include adults vs. children/adolescents, Socio-Economic Status, men vs. women, gender diverse vs. gender conforming)
Outcomes of interest	Social anxiety measures
Type of studies	Longitudinal studies, naturalistic observation studies, retrospective studies, prognostic studies, prevalence studies, repeated cross-sectional studies
Aim 2: How COVID-19 has affected individuals with a clinical diagnosis of Social Anxiety Disorder	
Population/Condition	Individuals with a diagnosis of Social Anxiety Disorder or who self-identify as having Social Anxiety Disorder
Interventions	Onset of the COVID-19 pandemic
Comparator	Comparators may include Individuals with no diagnosis of Social Anxiety Disorder
Outcomes of interest	All patient-oriented mental health outcomes
Type of studies	Comparative studies, longitudinal studies, naturalistic observation studies, retrospective studies, repeated cross-sectional studies, prognostic studies
Aim 3: Risk and protective factors that may have influenced levels of social anxiety in the context of the COVID-19 pandemic	
Population/Condition	All individuals affected by the COVID-19 pandemic
Interventions	Onset of the COVID-19 pandemic
Comparator	Individuals with differential scores on outcomes related to risk and protective factors
Outcomes of interest	Social anxiety measures
Type of studies	All study designs
Aim 4: How social anxiety has influenced affective, behavioural, and cognitive responses to the COVID-19 environment.	
Population/Condition	Individuals experiencing social anxiety during the pandemic
Interventions	Not Applicable
Comparator	Individuals with differential scores on social anxiety
Outcomes of interest	All patient-oriented mental health outcomes
Type of studies	All study designs
